# The embodied dynamics of perceptual causality: a slippery slope?

**DOI:** 10.3389/fpsyg.2015.00483

**Published:** 2015-04-21

**Authors:** Michel-Ange Amorim, Isabelle A. Siegler, Robin Baurès, Armando M. Oliveira

**Affiliations:** ^1^CIAMS, Univ Paris-Sud, Université Paris-SaclayOrsay, France; ^2^Centre de Recherche Cerveau et Cognition, Université de Toulouse, UPSToulouse, France; ^3^Centre National de la Recherche ScientifiqueCerCo, Toulouse, France; ^4^Institute of Cognitive Psychology – Faculty of Psychology and Educational Sciences, University of CoimbraCoimbra, Portugal

**Keywords:** causality, friction, embodied cognition, event perception, prediction

## Abstract

In Michotte's launching displays, while the launcher (object A) seems to move autonomously, the target (object B) seems to be displaced passively. However, the impression of A actively launching B does not persist beyond a certain distance identified as the “radius of action” of A over B. If the target keeps moving beyond the radius of action, it loses its passivity and seems to move autonomously. Here, we manipulated implied friction by drawing (or not) a surface upon which A and B are traveling, and by varying the inclination of this surface in screen- and earth-centered reference frames. Among 72 participants (*n* = 52 in Experiment 1; *n* = 20 in Experiment 2), we show that both physical embodiment of the event (looking straight ahead at a screen displaying the event on a vertical plane vs. looking downwards at the event displayed on a horizontal plane) and contextual information (objects moving along a depicted surface or in isolation) affect interpretation of the event and modulate the radius of action of the launcher. Using classical mechanics equations, we show that representational consistency of friction from radius of action responses emphasizes the embodied nature of frictional force in our cognitive architecture.

## Introduction

“It was a slippery, slippery, slippery slope

I feel me slipping in and out of consciousness”

Thom Yorke (2006) *Harrowdown Hill* lyrics excerpt

We inhabit a world where friction is omnipresent and crucial in our everyday life actions. Consider a world without friction (i.e., full of slippery surfaces): we could not walk without the friction between our shoes and the ground; neither could we hold any object (e.g., a pencil). When walking, our foot pushes backwards on the ground and the reaction force pushes us forwards thanks to friction. Actually, we often experience friction as a source of effort, such as when rearranging the furniture in a room. There is strong evidence that actions in our world, and the accompanying forces we experience, influence visual perception of distance and motion (Proffitt, 2006), and are at the origin of our causal understanding (White, 2006, 2009). For example, apparent distance or terrain inclination increases when wearing a heavy backpack or throwing a heavy object (Proffitt, 2006), and because our brain predicts the sensory effects and causal effects of our actions we can learn to play drums or basketball, but can hardly tickle ourselves (Blakemore et al., 2000). However, the mastery of physical forces accompanying our actions (e.g., playing music or sport) stands in sharp contrast to our poor explicit declarative knowledge about their underlying dynamics (McCloskey, 1983; Hecht and Bertamini, 2000).

Perception of collision events offers a variety of paradigms to unravel the structure of our mental representation of physical principles, e.g., perception of objects bouncing on the ground (Twardy and Bingham, 2002); control of rhythmic ball bouncing (Siegler et al., 2010), etc. Michotte (1963) and others (e.g., Schlottmann and Anderson, 1993; Schlottmann et al., 2006) showed that when an object A moves toward an initially stationary object B, and B is set into motion when A reaches B (and in turn A becomes stationary), the impression of A actively launching B does not persist beyond a certain distance identified as the “radius of action” of A over B (Yela, 1954; Boyle, 1961). While the launcher (object A) seems to move autonomously, the target (object B) seems to be displaced passively. This distinction is reminiscent of Newton's definition of force as both an action (*vis impressa* or impressed force) and also as a property of motion (*vis inertiae* or force of inertia). If the target keeps moving beyond the radius of action, it loses its passivity (*vis inertia*) and seems to move autonomously (*vis impressa*). Our contention is that measurement of the radius of action (RA) provides insight into the perceived kinetic properties of the event (Sinico and Parovel, 2002).

The notion that our understanding of dynamics stems from our experiences of acting on objects has been argued to offer a unifying account of visual impressions of forces, imagery implicated in the simulation of dynamic events, and explicit judgments about forces (White, 2009, 2011). This view assigns a unique role to proprioception and mechanoreceptors in the way forces are perceived and representationally construed. In contrast to claims of a direct perception of causality (Michotte, 1963), or of a direct specification of dynamics by kinematics (Runeson and Frykholm, 1983), it stipulates that visual impressions of force arise through the coupling of visual input with a knowledge base of embodied dynamics (White, 2006, 2009, 2012c,d). Distinctions between force and resistance, active and passive, or cause and effect, which do not form part of mechanics (standing in violation of its third law), become thereby a part of our understanding of mechanical interactions, and give rise to visual impressions of causality (White, 2006). One entailed consequence is that haptically embodied representations do not presume an isomorphism with physical invariants—e.g., kinematic geometry (Shepard, 1984, 2001), spatio-temporal coherence (Freyd, 1987, 1993), Newtonian principles (Sanborn et al., 2013). While they correspond to a form of internalization, they may as well be described as an externalization of body dynamics (Hecht, 2001), with the consequence that both their internal and external consistency remain in every case a matter for inquiry (Hecht and Bertamini, 2000; White, 2012b). Evidence exists that which forces come into awareness, and how they are interpreted, depends on mental simulations driven by our embodied knowledge of dynamics: discrepancy from predictions (in a forward model of action) may thus bring into awareness a force otherwise unnoticed (White, 2009, 2012a).

Michotte's launching display is suggestive of an elastic collision (e.g., between steel or pool balls) where the momentum (and kinetic energy) of the launcher is entirely transferred to the target and the target would keep traveling at the same velocity as the launcher just before contact, onto a surface without friction. However, both the concept of RA and the fact that the causal impression is increased when the target moves at a reduced velocity relative to the launcher (Michotte, 1963; Schlottmann and Anderson, 1993), suggest that implied friction is in the eye of the beholder. In the present study, we manipulated implied friction by drawing (or not) a surface onto which the launcher and the target are traveling (which is usually not the case in causal displays), and by varying the inclination of this surface in a screen-centered (horizontal or diagonal) and earth-centered (vertical or horizontal screen) frame of reference. We expected that the radius of action would vary with inclination of the drawn surface when objects are displayed on a vertical screen but not for a horizontal screen (seen from above). More precisely, due to implied gravity (Hubbard, 1990, 1997; Bertamini, 1993), RA would be greater for objects moving downwards (than upwards) onto an (screen-centered) inclined surface displayed on a vertical (earth-centered) screen. In contrast, when the animations are displayed on a horizontally-oriented screen, no effect of inclination is expected. Finally, we quantified the representational friction coefficient from RA responses using classical mechanics equations, in order to study the representational consistency of friction in causal displays, as a first approximation toward mental tribology (tribology is the science of interacting surfaces that are in relative motion, see Ludema, 1996). Consistency in the use of the coefficient of friction would follow if people acted according to physics (for the same pair of contacting materials, the coefficient of friction should remain the same across conditions). Inconsistency in its use, meaning a significant change in its estimated value across conditions, corresponds in turn to a deviation from what is entailed by physics.

While several meanings of embodiment co-exist in the psychology literature (Wilson, 2002; Shapiro, 2007; Wilson and Golonka, 2013), we take here a view of embodied cognition as the conjoining of two claims: that cognition is situated, on the one hand, and that “environmental invariants” (i.e., physical regularities in the world around us, such as gravity or friction) have been internalized in our cognitive system (Hubbard, 1995b), on the other hand. As an illustration of the first point, nobody expects equivalent collisional kinematics for a tennis ball dropped on the floor or rolling on the ground toward a wall: the ball will bounce several times on the floor, with observers having been shown capable of judging how natural the bouncing looks (Twardy and Bingham, 2002), whereas only once on the wall. Similarly, on the basis of our locomotor experience, we can expect moving furniture uphill to be more effortful than downhill. This is because we inhabit a world with gravity and friction that we sense with our body. As an illustration, now, of the second point, we do learn from experience that driving on a dry road is much less dangerous than on a wet or icy surface where friction is reduced and braking distance increases dramatically. On the basis of this knowledge, we can anticipate from weather forecast news the consequences for driving of impending poor weather conditions (e.g., need for reducing the speed and increasing the distance behind the vehicle in front by given amounts, depending on the particular route conditions and type of vehicle driven). Building on this 2-fold view, we examine here the contention that perception of collision events in a Michotte-type launching display will be driven by both environmental (i.e., orientation of the display with respect to gravity) and internal (e.g., coefficient of friction in prospective mental simulations of dynamical events) constraints resulting from embodied cognition.

## Experiment 1 (main experiment)

### Materials and methods

#### Participants

Fifty-two individuals took part in this experiment (mean age = 23 years, range = 19–34 years) after providing informed consent. They were divided in two different groups according to the experimental conditions. Local ethical approval from EA 4532 ethics committee of Université Paris-Sud was granted for this study.

#### Stimuli and apparatus

In the “friction” group, the launcher and target moved on a surface (thick gray line, 10 pixels wide) either horizontally, or on a diagonal trajectory downwards or upwards, −30° and +30° in screen-centered coordinates (*Motion slope* condition). In the “no friction” group, the launcher and the target motions were the same but without any surface displayed. The distance traveled by the launcher before entering in contact with the target was always the same in each trial (300 pixels). However, this launcher-target system could be initially positioned in three possible screen-centered coordinates (A, B, C) differing in 160 pixels steps along the motion path. The distance from the initial position of the launcher to the screen border in the direction of motion could be either 660, 500, or 340 pixels in the 0° *Motion slope*, and either 740, 580, or 420 pixels in the +30° and −30° (see sample Videos 1–7 in the Supplementary Material available online). The movies comprised 700 frames and lasted 7 s in total, with each frame played 10 ms. The motion could be rightward or leftward (mirror animations were used). The animations were 1024 × 768 pixels movies, and each square (launcher or target) side was 20 pixels. Because the launching effect is perceived as more natural with a velocity ratio of 3:1 for the launcher vs. target, respectively, (Michotte, 1963; Schlottmann and Anderson, 1993), that was also the velocity ratio we used in our displays. The launcher moved at 330 pixels/s (during the first 90 frames) whereas the velocity of the target was 110 pixels/s (approximately 3.3° of visual angle per second) after the contact.

**Figure 1:**
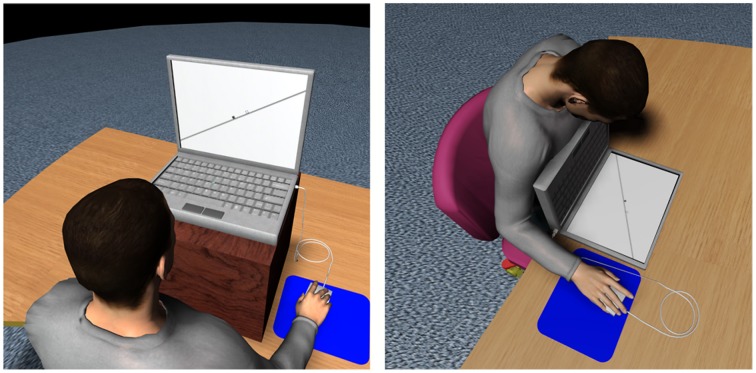
**Illustration of the vertical (left) and horizontal (right) *Motion plane* conditions, for objects traveling onto a +30° slope surface**.

The experiment ran on a HP Compaq nx9500 Laptop (17″ LCD screen) using ERTS-VIPL, a software package for programming psychology experiments (http://www.berisoft.com/). Depending on the *Motion plane* condition, participants faced either a vertical screen or a screen placed horizontally onto a table surface, from approximately 57 cm (see Figure 1). In contrast to *Motion slope* defined in a screen-centered (or viewer-centered) reference frame (referring to the configuration within the display), *Motion plane* is defined in an earth-centered frame of reference (referring to the orientation of the screen).

#### Procedure

In each movie, the launcher moved toward the target at constant velocity until it “collided” with the target. When the launcher reached the target, it stopped abruptly, and the target started to move in the same direction at reduced constant velocity (1/3 launcher velocity) until leaving the screen. It has been previously shown that such displays lead to induce strong launching impressions (Schlottmann et al., 2006) and that the motion of the launched object systematically appears passive (Parovel and Casco, 2006). While we did not ask participants to rate the strength of the perceived physical causality, we note that all participants during an exit debriefing mentioned having perceived the target to be launched.

After the target disappeared, a mouse cursor (in the form of a plus sign) was displayed in the center of the screen. Participants were required to place the mouse cursor over where the target square had been located when its motion was perceived to become “autonomous.” Participants were instructed that “autonomous” meant that the motion of the launched object was no longer passive, and were explicitly asked to match the center of the mouse cursor with the center of the target square. Note that the launcher was always displayed onto the screen until participants responded. The movie duration was the same whatever the *Motion slope* condition: 7 s. Therefore, although the distance from the ending screen border was 80 pixels shorter for the 0° condition as compared to the diagonal trajectories, the mouse cursor appeared always 6.1 s after the target started to move.

### Results and discussion

#### Statistical analyses of behavioral data

Repeated-measures ANOVAs on RA (in pixels) were conducted with *Implied friction* (friction vs. no-friction) as a between-subject factor, and with *Motion plane* (earth-centered: horizontal vs. vertical) and *Motion slope* (screen-centered: −30° vs. 0° vs. +30°) as within-subjects factors. *Motion direction* (whether leftward or rightward) as well as objects' *Initial position* (A, B, or C) were not considered in the statistical analyses. ANOVA, η^2^_*p*_ and Scheffé *post-hoc* tests were computed using SPSS 16.0 and Statistica 7, and the significance threshold was set to α = 0.05 unless otherwise specified. Means are reported together with 95% Confidence Intervals (see Table 1).

**Table 1:** **Descriptive statistics of behavioral data**.

***Implied friction***	***Motion slope***	**Radius of action (pixels)**		**Orthogonal displacement (pixels)**	
		***Motion plane* (earth-centered)**		***Motion plane* (earth-centered)**	
		**Vertical**	**Horizontal**	**Vertical**	**Horizontal**
Friction	+30°	139 (±31)	167 (±29)	−0.8 (±0.8)	−0.7 (±1.0)
	0°	177 (±30)	173 (±26)	−1.0 (±0.6)	−1.5 (±0.8)
	−30°	240 (±51)	192 (±34)	−0.1 (±1.2)	−0.8 (±1.1)
No-friction	+30°	225 (±43)	221 (±36)	0.4 (±2.5)	−1.2 (±1.6)
	0°	232 (±36)	224 (±30)	−1.5 (±0.8)	−1.0 (±0.6)
	−30°	253 (±43)	238 (±36)	−0.3 (±2.1)	−0.5 (±1.8)

*NB. Mean RA and O-displacement as a function of each condition (±95% CI)*.

First, the ANOVA showed a significant *Implied friction* effect with shorter RA in the condition of friction (*M* = 181 ± 27) than in the condition without friction (*M* = 232 ± 34), *F*_(1, 50)_ = 5.67, *p* = 0.021, η^2^_*p*_ = 0.10. The effect of *Implied friction* on RA varied with *Motion slope, F*_(2, 100)_ = 3.90, *p* = 0.023, η^2^_*p*_ = 0.07, but not with *Motion plane, F*_(1, 50)_ < 1. However, there was a *Motion slope* × *Motion plane* × *Implied friction* interaction, *F*_(2, 100)_ = 7.40, *p* = 0.001, η^2^_*p*_ = 0.13. In order to examine the latter interaction separate ANOVAs were conducted for each *Motion plane* or *Implied friction* condition.

**Figure 2:**
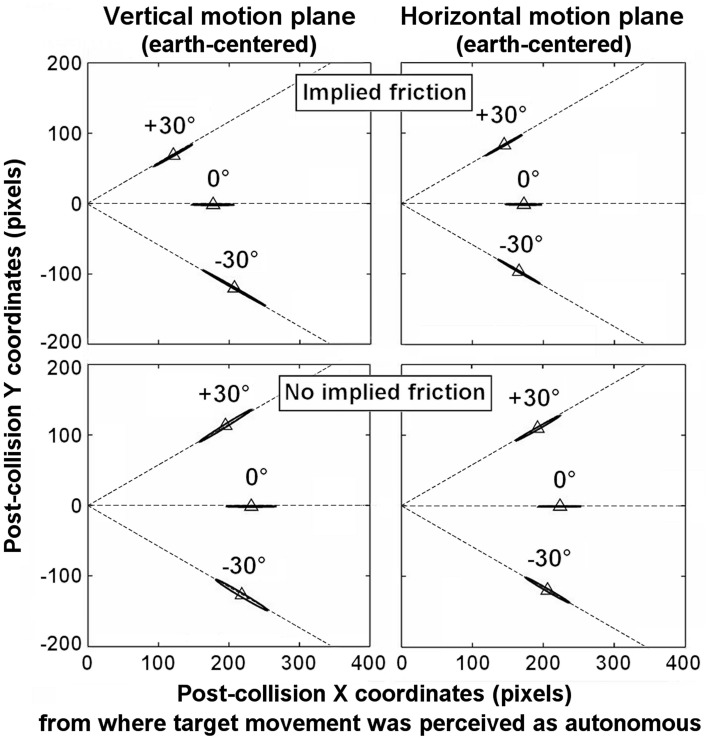
**Illustration of the 95% confidence interval ellipses around the mean position where participants felt the target movement started to be autonomous among each *Implied friction* group, for each condition of the *Motion slope* × *Motion plane* interaction**. The 0,0 coordinate in the left-hand y-axis on each panel reflects the initial location of the target (i.e., its location at the time it is contacted by the launcher). The dashed lines (not visible in the stimulus) illustrate the direction traveled by the target.

Separate ANOVAs for each *Motion plane* showed that the *Motion slope* × *Implied friction* interaction was significant in the vertical *Motion plane, F*_(2, 100)_ = 6.84, *p* = 0.002, η^2^_*p*_ = 0.12, but not for the horizontal *Motion plane, F*_(2,100)_ < 1. Moreover, *post-hoc* analyses confirmed that RA did not differ significantly between each cell of the *Motion slope* × *Implied friction* interaction for the horizontal *Motion plane* condition. In contrast, although RA did not differ significantly between the levels of *Motion slope* in the vertical *Motion plane* condition for the no-friction group, it presented differences in the friction group. More precisely, the RA for +30° was marginally (*p* = 0.065) smaller than for 0°, but significantly smaller than for −30° (*p* < 0.00001); and RA for 0° and −30° differed significantly (*p* < 0.000001). The patterns of means are available from both Figure 2 as well as Table 1.

Moreover, separate ANOVAs for each *Implied friction* group showed that the *Motion slope* × *Motion plane* interaction was significant for the friction group, *F*_(2, 50)_ = 13.51, *p* < 0.0001, η^2^_*p*_ = 0.35, but not for the no-friction group, *F*_(2,50)_ < 1. *Post-hoc* analyses for the no-friction group confirmed that RA did not differ significantly between each cell of the *Motion slope* × *Motion plane* interaction. In contrast, for the friction group, RA varied with *Motion slope* in the vertical *Motion plane* (RA for −30° was greater than for 0°, *p* < 0.00001, and for +30°, *p* < 0.00001), but not in the horizontal *Motion plane*. These differences in RA depending on *Implied friction* are visible in Figure 2 illustrating the 95% confidence interval ellipses (computed using the student's *t* distribution) around mean response for each cell of *Motion slope* × *Motion plane*. The 0,0 coordinate in the left-hand y-axis on each panel refers to the initial location of the target (i.e., its location at the time it is contacted by the launcher).

Finally, in order to control that the RA did not vary due to spatial memory displacement away from the target trajectory (Hubbard, 1997), especially in the no-friction group where the motion surface was not displayed, we computed the orthogonal distance between participants' response and the target trajectory (“trajectory” meaning the path of motion of the target center). Orthogonal distance is a measure of deviation from the target path combining the x and y distances $\left(\sqrt{\left({\text{x}}^{2}+{\text{y}}^{2}\right)}\right)$ between the point indicated by the participant and the point in the true path of motion along a line passing by the center of the target and intersecting the true trajectory at an angle of 90°. A measure of positive or negative O-displacement was then associated to this distance, depending on whether the indicated point was above or below the path of motion in screen-centered coordinates, respectively (for the original distinction between M- and O-displacements, see Hubbard, 1995a). Table 1 shows negligible mean O-displacements (within the range of target's height), and the ANOVA showed neither main effects of the experimental factors nor interactions on O-displacement values, which suggests that variations of RA are not related to spatial memory displacement orthogonal to the target trajectory.

### Physical modeling of friction from radius of action responses: friction coefficients computation

The friction coefficient μ is a dimensionless scalar value which describes the ratio of the force of friction between two bodies and the force pressing them together. The coefficient of friction depends on the materials used; for example, ice on steel has a low coefficient of friction, while rubber on pavement has a high coefficient of friction. Coefficients of friction range from near zero to greater than one. A friction coefficient μ = 1 means that the force needed to move the object is equal to its weight, <1 that it is less than its weight, and >1 that it is larger than its weight. For example, under good conditions, a tire on concrete may have a coefficient of friction of 1.5 (Ludema, 1996). In order to study the representational consistency of friction in our causal displays, we computed the (representational) friction coefficient from radius of action responses using classical mechanics equations.

The subjective value of friction coefficient μ for the target was computed with the equations below under the assumption that the target will decelerate post-collision due to friction, with the following parameters: object mass *m*; gravity acceleration g is a constant (9.81 ms^−2^); target's deceleration *dec* (<0) after collision as inferred from participant's response (see below); and the slope angle Θ. Note that actually μ does not depend on *m*. This is because *m* appears as a factor in both terms of the fraction and therefore can be canceled off, and the fraction simplified.

(1) $$\mu =\frac{-m.dec-m.g.\sin\Theta }{m.g.\cos\Theta }=\frac{-dec}{g.\cos\Theta }-\tan\Theta$$

**Figure 3:**
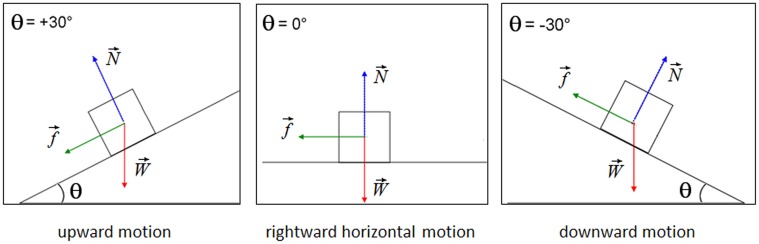
**Illustration of the forces concomitant to a rightward motion**.

To calculate the forces on an object placed on an inclined plane (with slope angle θ), one must consider the three forces acting on it (air resistance is neglected for the sake of simplicity), as illustrated in Figure 3:

1. The force $\vec{W}$due to gravity (the object's weight “mg,” acting vertically downwards)
2. The normal force ($\vec{N}$) exerted on the body by the ground surface, perpendicular to the surface, in reaction to $\vec{W}$exerted on the ground and
3. The frictional force ($\vec{f}$) acting parallel to the ground surface, always exerted in a direction that opposes movement (for kinetic friction) or potential movement (for static friction) between the two surfaces. When pushing an object, as long as the object is not moving, the magnitude of the force of static friction equals that of the applied force. When this force exceeds the magnitude of the force of kinetic friction, the object is put into motion (it accelerates).

We assumed that the position indicated by participants reflects the moment (time Δ*t*) when they detected a difference Δ *V* between the predicted (on the basis of a representational friction coefficient and an analogy to the physics of friction) vs. perceived velocity (taken to be approached by the actual velocity) of the target, as indicated in the instructions: “*the movement of the target seems to be autonomous*.” Δ*t* was inferred from participant's response (mouse click on XY screen coordinates: *Xobs, Yobs*) with Equation (2) where *Vobs* stands for the actual target's velocity on the screen (110 pixels per second).

(2) $$\Delta t=\frac{\sqrt{\left(Xobs^{2}+Yobs^{2}\right)}}{Vobs}$$

Because literature indicates that our visual system detects a change in velocity when greater than 25% of actual velocity (e.g., Calderone and Kaiser, 1989; Babler and Dannemiller, 1993), we assume that participants detected a change in target velocity *Δ V* when it reached a threshold value expressed as a percentage of actual target velocity *Vobs*. Thus, Equation (3) expresses Δ *V* as a function of a coefficient *k*, with *k* = 0.25. Furthermore, in order to express Δ *V* in meters per second, the self-to-scene subjective distance *D_scene_* (i.e., subjective distance to the objects, which has to be estimated from participants' responses) and self-to-screen distance (*D_Screen_* = 0.57 m) had to be taken into account:

(3) $$\Delta V=k.Vobs\times \frac{D_{Scene}}{D_{Screen}}$$

This stems from the circumstance that the moving square can either be perceived as the motion of an object on the screen plane or as the projection of a distant moving object on that plane; in the two cases, the visual angle is the same. Since the physics model and calculations of μ require to express the kinematics in physical units (m, m/s, m/s^2^), this self-to-scene distance factor had to be factored in the model and computations.

Equation (4) provides the predicted target's subjective deceleration as a *negative variation of the predicted target velocity* over *time*.

(4) $$dec=-\frac{\Delta V}{\Delta t}=-\frac{k.Vobs}{\Delta t}\times \frac{D_{Scene}}{D_{Screen}}$$

Vertical plane (earth-centered) condition with implied friction for objects traveling on a horizontal surface (Θ = 0°) served as a theoretical reference condition. Setting μ = 1 for this condition allowed us to examine the variation of μ in the other conditions, in proportion to this 0° reference condition. In this reference condition, with μ = 1, Eq. 1 yields *dec* = –*g*. In order to compute μ for the other conditions using Equation (4) and (1), we first computed the average subjective *D_scene_*-value for the reference condition, using Equation (5) derived from Equation (4).

(5) $$D_{Scene}=g.D_{Screen}\times \frac{\Delta t}{k.Vobs}$$

In brief, the strategy of our approach consisted in using a behavioral measure of RA, via a set of classical mechanics equations and some identified reasonable assumptions, to compute the representational equivalent of a “friction coefficient.” The key assumption in the process is that, in order to comply with the instructions to locate where the target motion became autonomous, participants rely on the detection of a discrepancy between a predicted diminished velocity of the target, reflecting an expected deceleration due to friction, and the actual observed velocity (Δ *V*). The deceleration expected by participants was then derived from the ratio between Δ *V* and the time when they detected this difference (as inferred from RA responses). From this deceleration value, a representational friction coefficient μ was computed using Equation 1. After setting the theoretical reference value to μ = 1 in the vertical *Motion plane* condition for objects traveling along a 0° *Motion slope* visible surface (friction group), we proceeded to compare the μ values of the other conditions to this reference value after Bonferroni correction (α = 0.05/11).

**Figure 4:**
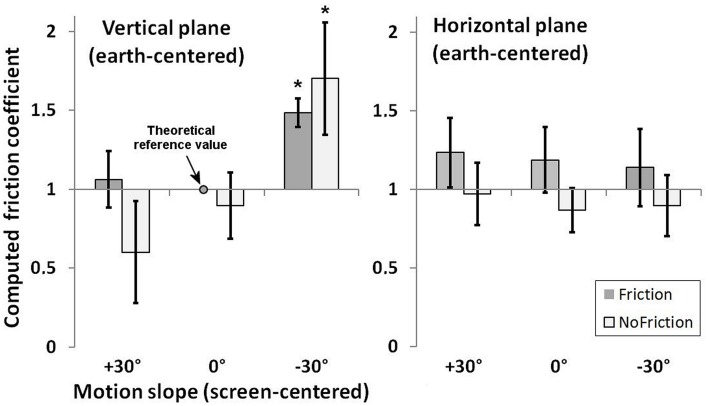
**Computed friction coefficients (μ) for each condition after setting the theoretical reference value to μ = 1 in the vertical *Motion plane* condition for objects traveling along a 0° *Motion slope* visible surface**. Stars indicate significant departure from the theoretical value after Bonferroni correction.

Results, illustrated in Figure 4, show similar values of the representational friction coefficients in the horizontal *Motion plane* for each *Implied friction* group. In contrast, in the vertical *Motion plane*, the friction coefficient varied with *Motion Slope* and *Implied Friction* conditions. The variation of the representational friction with slope for the no-friction group results from the invariance of RA across *Motion slope* conditions, which would be unexpected from the standpoint of physics. As a consequence, if friction was to explain this invariance, the friction coefficient would have to be smaller for ascending slopes and greater for descending slopes, as compared to 0°. Such representational inconsistency of friction seems unreasonable and suggests rather that participants of the no-friction group reasoned about object motion independently of environmental invariants, such as gravity or friction. Finally, the RA estimate of the friction group in the vertical *Motion plane* condition is in agreement with physics for the ascending slope, whereas it is not for the descending slope where the representational friction is greater than the reference value, *t*_(25)_ = 10.93, *p* < 0.05/11, Cohen's *d* = 2.14 (see Figure 4).

Friction from the ground-object contact acts as a brake that dissipates kinetic energy from the moving objects into thermal energy. Interactive Physics 2000© allowed us to simulate this dissipation along time for the launcher and the target, for equivalent launcher velocity just before contact, 1 kg objects and μ = 1 (see Videos 8–10 in the Supplementary Material available online). Remember that, as mentioned above, μ does not depend on *m*. However, the mass of each object had to be specified in order for the software to perform the simulations. Contrary to Michotte's displays, simulated motion is that of an inelastic collision (some of the initial kinetic energy is lost while converted into internal excitation, heat, or eventually into deformation) with decreasing object velocity due to friction. Both the launcher and the target continue to move after collision to a different extent depending on motion slope. Actually, the traveled distance after collision varies as a function of *Motion slope* with a pattern resembling that of the RA estimates of the friction group in the vertical *Motion Plane* condition. However, if RA reflects expected stopping point, the estimate for the descending slope is smaller than expected according to physics (mean RA for −30° is about 1.36 greater than for 0°, whereas Interactive Physics 2000© simulations indicate a target traveled distance about 2.82 greater for −30° as compared to 0°), suggesting an increase in the representational friction as compared to the 0° reference condition. In other words, the observed RA on the −30° condition would have to be larger to agree with the physical model (with an invariant friction coefficient μ = 1). For explaining through friction the smaller ratio of the observed RA in the −30° with respect to 0° conditions, the physical model would imply an increase in the “friction coefficient.”

In order to test if the increased representational friction for −30° might result from a low-level effect due to the proximity of the screen that would impose a limit to RA responses we performed an additional ANOVA on RA while introducing *Initial position* (A, B, or C) as a supplementary within-subject factor. Results indicated that although there was a main effect of *Initial position* on RA (*M*_A_ = 219 ± 26; *M*_B_ = 206 ± 23; *M*_C_ = 195 ± 20), *F*_(2, 100)_ = 19.05, *p* < 0.000001, η^2^_*p*_ = 0.28, this factor did not interact with any of the other three factors. Therefore, the initial target distance to the screen border had an overall effect not specific to descending slopes. The observed decrease of RA with initial distance of the target from the screen border was negligible (range = 13–19 pixels, i.e., smaller than the target size) when initial distance to the screen border decreased by 160 pixels steps between *Initial position* A and B or B and C. Finally, although we did not formulate any hypothesis about an effect of *Motion direction* (whether leftward or rightward), we ran an ANOVA including both *Initial position* and *Motion direction* in addition to previous factors, as a further control to make sure that counterbalancing factors did not matter. As it turned out, *Motion direction* was not significant, *F*_(1, 50)_ = 2.99, n.s., η^2^_*p*_ = 0.06, nor did *Motion direction* interact with *Initial position, F*_(2, 100)_ = 1.83, n.s., η^2^_*p*_ = 0.04. Moreover, regarding the *Motion slope* × *Motion plane* × *Implied friction* interaction of interest, neither *Motion direction, Initial position*, nor both taken together interacted with it, *F*s_(2,100)_ < 1.

## Experiment 2 (control experiment)

The validity of our friction coefficient computations rests on the assumption that RA provides a measure of the time when observers detected a difference between the predicted vs. actual behavior of the target. In order to ascertain this hypothesis we performed a control experiment where participants indicated this time point on-line vs. a posteriori.

### Materials and methods

#### Participants

Twenty individuals took part in this experiment (mean age = 20 years, range = 18–27 years) after providing informed consent. None of them had participated in Experiment 1. Local ethical approval from EA 4532 ethics committee of Université Paris-Sud was granted for this study.

#### Stimuli and apparatus

Stimuli and Apparatus were similar to those used for the friction group in the Experiment 1.

#### Procedure

Participants indicated the position/moment from which they felt that “the target movement started to be autonomous” either a posteriori with the mouse cursor (as in the Main Experiment) or on-line by pressing the space bar. These *Response type* conditions were performed in two separate counterbalanced blocks. Moreover, participants were tested only in the vertical Motion plane with friction.

### Results and discussion

**Figure 5:**
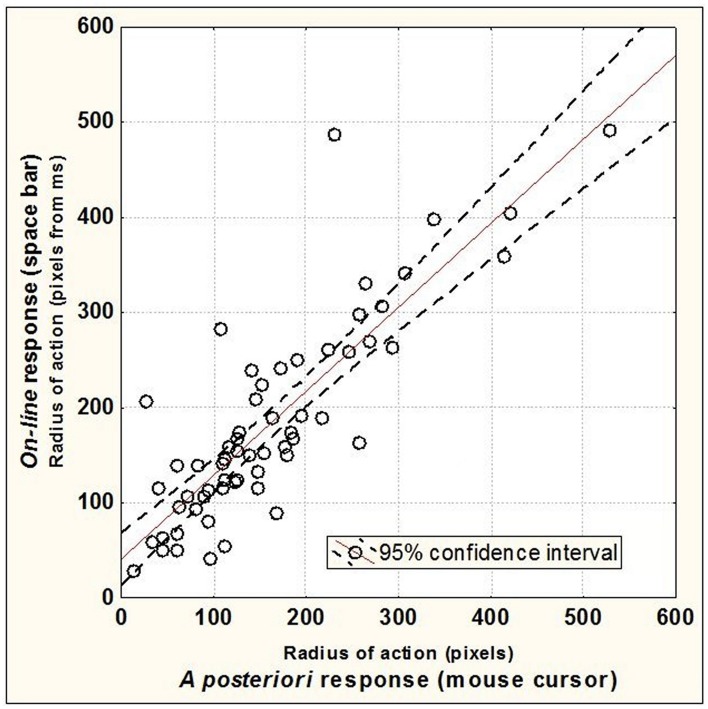
***On-line* Radius of Action as a function of *A posteriori* Radius of Action, for each participant; each point corresponds to individual mean data for a given level of the *Motion Slope* condition (three data points per participant)**.

Time data (in ms) of the *on-line* response condition were converted into RA values (in pixels). A simple regression was performed in order to test if spatial RA of *a posteriori* responses predicted temporal RA of *on-line* responses (see Figure 5). The linear regression model (*Y* = 12.54 + 0.81X) predicted significantly the data with a significant slope [β = 0.84, *t*_(58)_ = 12.09, *p* < 0.000001] and an intercept not different from zero [*t*_(58)_ = 0.88, n.s.]. These results are consistent with our hypothesis that spatial RA provides a good approximation for the time point when observers detected a difference between the predicted vs. actual behavior of the target.

**Figure 6:**
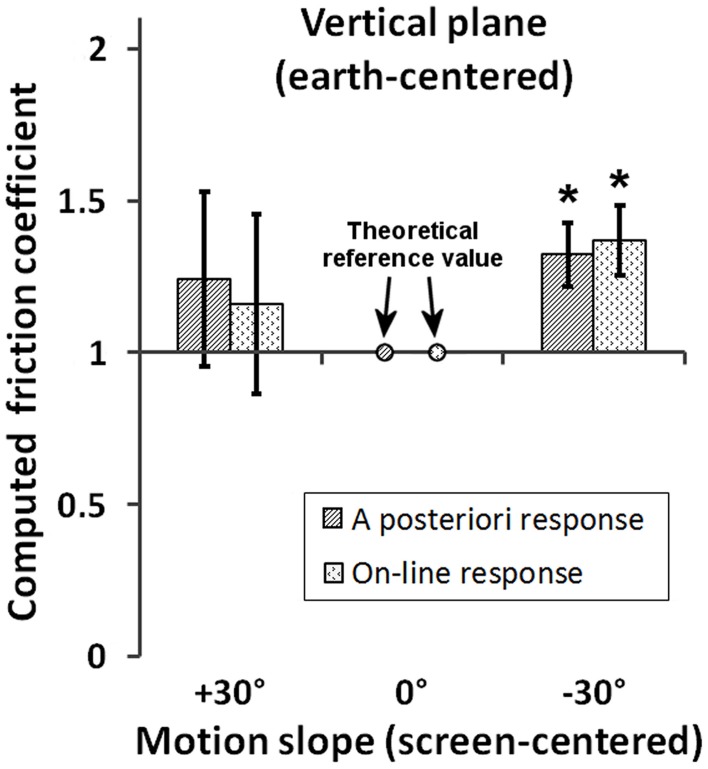
**Computed friction coefficients (μ) for each *Response type* condition (on-line vs. a posteriori) and *Motion slope* condition in the vertical *Motion plane* with implied friction**. The theoretical reference value was set to μ = 1 for objects traveling along a 0° *Motion slope* visible surface. Stars indicate significant departure from the theoretical value after Bonferroni correction.

Furthermore, in order to see whether results in Experiment 1 replicate in the vertical plane, friction condition, we computed representational friction coefficients after setting the theoretical reference value to μ = 1 in the vertical *Motion plane* condition for objects traveling along a 0° *Motion slope* visible surface. For each *Response type* condition, the resulting mean μ-values for the +30° and −30° *Motion slope* conditions were compared to this reference value after Bonferroni correction. In addition to these four comparisons we also tested if the μ-values for +30° and −30° differed between *Response type* conditions. Therefore, the Type I error threshold was set to α = 0.05/6. Results, illustrated in Figure 6, indicated that representational friction coefficients differed from the reference only for −30° for both the *on-line* [μ = 1.35, *t*_(19)_ = 6.53, *p* < 0.05/6, Cohen's *d* = 1.46] and *a posteriori* responses [μ = 1.32, *t*_(19)_ = 6.34, *p* < 0.05/6, *d* = 1.42], thus replicating the findings of Experiment 1. In addition, mean μ-values did not differ between *Response type* conditions neither for the +30° motion slope [*t*_(19)_ = 0.88, n.s., *d* = 0.20], nor for the –30° [*t*_(19)_ = 0.91, n.s., *d* = 0.20] conditions.

Taken altogether, the empirical elements available (regression model and friction coefficients) speak in favor of the soundness of using the spatial measure of RA as a reasonable approximation to the moment when the participants detect a difference between predicted and observed velocity, and thus as a means for the estimation of “friction coefficients.”

## General discussion

In spite of variations in surface layout or in friction in the world we inhabit, we seldom fall or slip in our adult lifetime, especially when standing or walking on (earth-centered) horizontal surfaces. However, when considering potentially slippery slopes for unfamiliar surfaces, we tend to rely more on visual than tactile information regarding friction (Joh et al., 2007). As a consequence, we explicitly overestimate our locomotor or standing ability on low-friction surface (e.g., vinyl), and underestimate our ability on high-friction surfaces (e.g., rubber). In contrast, since the brain can predict the consequences of the forces we generate (e.g., to adjust grip force when striking a ketchup bottle to prevent it from slipping (Wolpert and Flanagan, 2001) and experience (e.g., when returning a tennis ball), relations among forces appear to be internalized to some extent. Here, we used computer-generated displays based on Michotte's causal displays to investigate to what extent mental representations of collision events preserve similarity relations with properties of physical invariants (viz., second-order isomorphism, (Shepard and Chipman, 1970). For this purpose, we quantified the representational friction coefficient from radius of action (RA) responses to Michotte's causal displays using classical mechanics equations, in order to identify how representational consistency of frictional force in collision events is modulated by contextual information.

We reasoned that if physical invariants are embodied in the cognitive architecture, implied friction will affect RA with a pattern depending on the orientation of the display with respect to environmental constraints such as gravity direction. Because friction is proportional to the magnitude of the normal force acting on the object, which depends on the orientation of the support surface with respect to gravity, if cognition is situated then implied friction should be instantiated differently as a function of (viewer-centered) motion slope and (earth-centered) motion plane. Literature provides several examples where embodiment of gravity via proprioception influences visual perception and our prediction of motion: whatever observer's orientation with respect to gravity, tunnel turns are perceived more bent when the end is pointing in the direction of gravity (Vidal et al., 2006); virtual objects moving at constant velocity in the direction of gravity induce earlier interceptive responses (Senot et al., 2005) and greater spatial memory displacement (Nagai et al., 2002) than when moving in the direction opposite to gravity.

First, we found that when the support surface on which both launcher and target are traveling is not explicitly displayed, RA did not vary as a function of motion slope nor motion plane, as if the representational friction (resistive force) was constant across orientations. Moreover, participants were very consistent in their response as indicated by the insignificant orthogonal deviations from the target path. At first glance, this might suggest that when the support surface is not part of contextual information, participants would reason about object motion independently of environmental invariants, just as young infants believe that self-propelled objects require no external support to move in midair (Luo et al., 2009). However, on the one hand, invariance of RA across motion slope in the horizontal motion plane might be compatible with implied physics, since the gravity direction is constant across motion slope, which thus suggests some internal consistency with respect to the earth-centered referential. On the other hand, the similar invariance found for the vertical motion plane appears more consistent with an impetus heuristics (Kozhevnikov and Hegarty, 2001). In the more influential versions of the impetus theory, the imparted impetus that sets a body in motion in a given direction has to fully dissipate or to be strongly diminished for gravity to exert an effect and eventually prevail. This framework would thus lead to predict invariance of the RA across motion slope as long as dissipation of the imparted impetus didn't reach the required point for gravity to be factored in. In the vertical motion plane, such invariance is definitely inconsistent with motion along an invisible surface with a constant friction coefficient across motion slope.

When both launcher and target are traveling in sequence along a path displayed in the visual scene, the effect of this implied surface varies as a function of motion plane. RA is invariant across motion slope when motion plane is horizontal; however, mean RA is smaller by a constant amount (about 2 times an object size) than when no implied surface is displayed. This suggests that object's motion was interpreted as an instance of wall hugging, resulting in an expected overall reduction of the target's velocity by virtue of friction, while the computed friction coefficient still did not differ from the reference condition (objects moving along a horizontal slope surface in the vertical motion plane). As for objects' motion in the vertical plane, RA varied as a function of motion slope in a way roughly consistent with the effect of representational friction being modulated by gravity, i.e., smaller RA for ascending slopes and greater RA for descending slopes (as compared to 0°). However, although there was internal consistency between the representational friction coefficients for ascending and horizontal slopes, the computed friction coefficient for descending motion was 1.5 greater than for the two other conditions. One possible conjecture consistent with this result is that the increased friction coefficient for descending targets would reflect an embodied braking of the target. Similar to experimental participants who project their intention to throw objects and mistakenly believe that objects would initially continue to accelerate shortly after leaving their hand (Hecht and Bertamini, 2000), our participants might have embodied the target to simulate braking in order to rapidly reach the deceleration required to stop the target safely (Fajen, 2005). This interpretation is consistent with authors arguing that our actions in the world are the origin of our visual impressions of force between interacting objects (White, 2009), and that environmental constraints are perceptually scaled to the economy of action (Proffitt, 2006). For example, when we descend hills it requires earlier/greater braking to counteract the gravity pull. Similarly, walking on a slippery floor is more likely to lead to falls and consequently needs to be carefully handled.

Recognition of surface slipperiness has been embodied in our perceptual system through experience. Although visual and auditory cues to slipperiness are less informative than sliding resistance from tactile cues (Cohen and Cohen, 1994), subjective slipperiness ratings made from available visual cues (such as reflectiveness and texture) are consistent with actual coefficients of friction of surfaces (Lesch et al., 2008). This remains true even among vision-impaired elderly persons, though to a lesser extent (Hsu, 2011). Our brain in-builts bisensory (haptics and vision) texture-selectivity regions located in the medial occipital (Stilla and Sathian, 2008) and medial occipitotemporal (Podrebarac et al., 2014) cortices. This suggests literally that we may in some sort “feel” (haptically-like) sliding resistance from the textures we see (White, 2012b). Visual features of a surface, such as the thick gray line on/aside which the launcher and target moved in our experiment (see Figure 1), may be used to elaborate a mental image of its roughness (Newman et al., 2005) and in turn predict the forces encountered by the launched object in a Michotte's display. Such visuo-haptic mental simulation process would modulate the radius of action of a launcher over a target.

Our data suggested that the representational friction affects the radius of action of a launcher over a target in Michotte-type displays, with a pattern reflecting the embodiment of physical invariants in the cognitive architecture. These findings run counter Michotte's statement, directed against the projective interpretation of visual events, that only the visual structure of the event can cause a fusion between kinaesthetic and visual impressions (Michotte, 1941, p. 123–124). Here, we show that both physical embodiment of the event (looking at a vertical vs. horizontal plane, cf. Figure 1) and contextual information (objects moving along a surface) affect interpretation of the event and modulate the radius of action of the launcher. Literature also provides similar evidence of embodied friction. Objects sliding along a surface exhibit less forward displacement in memory (representational momentum) than when moving in isolation (Hubbard, 1998), due to the representational friction (Hubbard, 1995a); representational momentum is even more reduced when the object moves between two surfaces. In addition to replicating the general findings of greater representational friction for objects sliding along a surface than when moving in isolation, we provide quantitative estimates of representational friction coefficients, in order to test the consistency of their embodiment (internalization through experience) in our cognitive architecture. Moreover, we provide evidence that mental simulation of resistive force is situated, as implied friction varied with orientation of the display with respect to gravity in an earth-centered rather than eye-centered frame reference frame. Our results go along the lines of a series of experiments by Senot and colleagues (Senot et al., 2005, 2012; Le Seac'h et al., 2010) showing earlier interceptive responses for catching a virtual ball falling from “above” (as compared to approaching from “below”) in alignment with respect to the gravity pull, but not when looking to the same scene (with visual “up” and “down”) orthogonally to gravity (Senot et al., 2005). In our study, body posture varied while looking down to a horizontal display versus straight ahead to a vertical display. However, with experiments run on Earth or weightlessness (parabolic flights), Senot et al. showed that the only important parameter is the orientation of the moving object with respect to the gravity pull (sensed by our otolith receptors), and not the body posture (Le Seac'h et al., 2010; Senot et al., 2012). Similarly, the up/down asymmetry in estimating the pitch angle of a virtual corridor traveled on Earth reduces dramatically while free-floating in weightlessness (International Space Station). This asymmetry may be restored by attaching astronauts with belts and foot straps providing haptic inputs that help the brain reconstruct the missing gravitational cues (De Saedeleer et al., 2013). In summary, sensing gravity's pull (by the otoliths or contact forces) appears as the main parameter for cognition to be situated and, in our case, simulating the forces exerted on a launched target moving along a surface.

The embodiment of both friction and gravity forces in our cognitive architecture may well be more pervasive than we may at first think. Embodied theories of conceptual representation propose that the human sensorimotor system may serve to embody abstract ideas and metaphors (Lakoff and Johnson, 1999; Gallese and Lakoff, 2005). The gravity pull is “tightly linked” to our conception of emotion and morality. Positive words are associated to UP whereas negative words to DOWN (Lakoff and Johnson, 1999; Lakens, 2012), and both metaphorical associations automatically reactivate corresponding directional body movements (Koch et al., 2011) and postures (Dudschig et al., 2015). Multimodal simulation from language may also sustain *The Mind is A Body* and *Thinking Is Physical Functioning* metaphors (Lakoff and Johnson, 1999, p. 237) and explain why one might feel slipping in and out of consciousness just as on a slippery slope (see introductory excerpt), or why it is difficult “to resist the force of an argument” or “the overwhelming weight of evidence.” Similarly, while analyzing the mechanisms of slippery slopes arguments in the legislative-judicial domain, Volokh (2003) emphasized that “People resist attempts to take rights away outright, but not if the rights are eroded slowly.” In the latter metaphor, erosion supposedly alters coarseness of the surface, which in turn diminishes the resistive force of friction. In the present study, although roughness of surfaces was unspecified in the displays, the coefficient of friction emerged as a parameter in the mental simulations of participants seemingly driven by their embodied knowledge of dynamics.

## Conclusion

In Michotte's launching displays, if the target keeps moving beyond the radius of action (RA) of the launcher (over the target), the target loses its passivity and seems to move autonomously. Our findings show that when both launcher and target are traveling along a thick gray line displayed in the visual scene, the effect of this implied surface on the moment when the target movement is perceived as autonomous reveals how friction is embodied in our cognitive system. For objects' motion in the vertical plane, RA varied as a function of motion slope in a way roughly consistent with the effect of representational friction being modulated by gravity. In contrast, when the surface on which both launcher and target are traveling was not explicitly displayed, RA did not vary as a function of motion slope nor motion plane, as if the representational friction from the milieu was constant across orientations, which is more consistent in turn with an impetus heuristics interpretation. Therefore, the possibility remains that embodied knowledge of forces (whether exerted or resistive) gives raise to scattered concepts or micro-theories which mediate judgments about force (Hecht, 2001) and make up distinct branches of expression of a bodily-based dynamics (White, 2012b,c). When studying perceptual causality, not keeping in mind the role of embodied dynamics and the potential heterogeneous coexistence of resulting micro-theories in our cognitive system is what might well turn out being a slippery slope at the end.

### Conflict of interest statement

The authors declare that the research was conducted in the absence of any commercial or financial relationships that could be construed as a potential conflict of interest.

## Supplementary material

Video 1 **This movie illustrates a rightward 0° *Motion slope* with implied friction from *Initial position* A**.

Video 2 **Idem from *Initial position* B**.

Video 3 **Ibidem from *Initial position* C**.

Video 4 **Rightward +30° *Motion slope* with implied friction from *Initial position* B**.

Video 5 **Idem but for a −30° Motion slope**.

Video 6 **Leftward +30° *Motion slope* without implied friction from *Initial position* B**.

Video 7 **Idem but for a rightward motion**.

Video 8 **This movie shows a friction simulations from the Interactive Physics 2000© software for the vertical *Motion plane* condition and +30° *Motion slope***.

Video 9 **Idem for a 0° *Motion slope***.

Video 10 **Ibidem for a −30° *Motion slope***.
